# CD16 expression on neutrophils predicts treatment efficacy of capecitabine in colorectal cancer patients

**DOI:** 10.1186/s12865-020-00375-8

**Published:** 2020-08-08

**Authors:** Yu Lu, Yizhou Huang, Lei Huang, Yanjie Xu, Zien Wang, Han Li, Ting Zhang, Ming Zhong, Wei-qiang Gao, Yan Zhang

**Affiliations:** 1 State Key Laboratory of Oncogenes and Related Genes, Renji-Med X Stem Cell Research Center, Renji Hospital, School of Medicine, Shanghai Jiaotong University, Shanghai, China; 2 Department of Gastrointestinal Surgery, Renji Hospital, School of Medicine, Shanghai Jiaotong University, Shanghai, China; 3 Med-X Research Institute & School of Biomedical Engineering, Shanghai Jiaotong University, Shanghai, China

**Keywords:** CD16, Neutrophils, Capecitabine-resistance, Colorectal cancer

## Abstract

**Background:**

Early detection of capecitabine-resistance could largely increase overall survival of colorectal cancer (CRC) patients. Previous studies suggested examination of immune cells in peripheral blood would help to predict efficacy of chemotherapy.

**Methods:**

We examined the immunological characteristics of peripheral blood in CRC patients with capecitabine treatment. We analyzed the relationships between the abnormal immune cell population in capecitabine-resistance patients and major clinical features. Furthermore, RNA sequencing, analyses of cell surface marker expression and the correlations with other major immune cell populations were performed using this population to explore the possible function of these cells.

**Results:**

The expression level of CD16 on neutrophils was down-regulated in capecitabine-resistant CRC patients. Patients with CD16^low/−^neutrophils after capecitabine therapy had adverse clinical features. What’s important, the change of CD16 expression level on neutrophils appeared much earlier than CT scan. RNA sequencing revealed that CD16^low/−^neutrophils in capecitabine-resistant patients had lower expression level of neutrophil-related genes, compared to CD16^+^neutrophils in capecitabine-sensitive patients, suggesting this CD16^low/−^population might be immature neutrophils. Furthermore, the expression level of CD16 on neutrophils in patients with capecitabine treatment was positively correlated with the number of anti-tumor immune cell subsets, such as CD8^+^T cell, CD4^+^T cell, NK cell and monocyte.

**Conclusions:**

Our findings indicated that CD16 expression on neutrophils in peripheral blood was a good prognostic marker for predicting efficacy of capecitabine in CRC patients.

## Background

Colorectal cancer (CRC) is one of the leading cause of death worldwide. More than 1.8 million patients are diagnosed with CRC every year [1–3]. What’s more, this life-threaten disease kills nearly 0.9 million people annually [1]. In north America and Europe, the morbidity and mortality remain at high level [1], despite developments of cancer screening and endoscopy [2, 3]. In China, CRC becomes the 5th most diagnosed cancer and 5th most deadly cancer [4–6]. Nearly 0.4 million new cases are diagnosed and about 0.2 million people die from the disease every year [6].

Postoperative adjuvant chemotherapy is first-line treatment for CRC patients [7, 8]. Capecitabine, a carbamate derivative of fluoropyrimidine, is the backbone of CRC chemotherapy [9, 10]. As the oral prodrug of 5-fluorouracil (5-FU), it is widely used for postoperative adjuvant chemotherapy due to its long, stable duration, lower toxicity and convenient dosing compared to infusional 5-FU [7, 11]. However, this chemotherapeutic drug has only modest efficacy, the response rates of 5-FU for advanced CRC is only 15% for single treatment and 50% for combined chemotherapy [12, 13]. The chemoresistance is recognized as a principal obstacle for cancer therapy [14–16], leading to tumor recurrence or metastasis, especially liver and lung metastasis, and cause over 90% of CRC mortality [17]. Intense researches on the mechanisms underlying the resistance revealed that changes of tumor cells themselves cause resistance, although these findings are mainly restricted to tumor specimen examine, which is not that suitable for post-treatment surveillance. What’s more, CT (computed tomography) scan and colonoscopy are insensitive to micro metastasis, despite their good accuracy for the detection of recurrence. Capecitabine-resistant patients could only be diagnosed with cancer recurrence by CT scan or colonoscopy about 2–3 years after capecitabine therapy [18], when tumors are big enough to be discovered. Thus, good prognostic markers are indispensable for predicting capecitabine-resistance in the early stage after capecitabine therapy.

Cancer cells and their microenvironment could interact with each other. Immune cells could dynamically reflect cancer status and display multifaceted functions in cancer development [19–22]. Myeloid cells, including monocytes, macrophages, granulocytes (neutrophils, eosinophils, basophils) and mast cells, play critical roles in cancer progression [19–22]. Myeloid-derived suppressor cells (MDSCs), a heterogeneous population of myeloid cells remain at different stages of differentiation, are immature counterparts of myeloid cells in cancer. MDSCs acquire immunosuppressive features and mainly inhibit lymphocytes, including T cells and NK cells [23–25]. Recent studies report that chemotherapeutic agents, like 5-FU, could interact with myeloid cells, and influence anti-tumor efficacy [26–29]. Vincent J et al. reported that 5-FU selectively induced MDSC apoptotic cell death and increase IFN-γ production by tumor-specific CD8^+^T cells [26]. Other researchers showed that activation of NLRP3 inflammasome and increased amount of HSP70 exosomes on MDSC by 5-FU lead to MDSC activation [27, 28]. Yuan Y et al. found that tumor-associated macrophages secret IL-6 to induce 5-FU chemoresistance [29].

In this study, we discovered that the expression of CD16 on CD11b^+^myeloid cells was dramatically decreased in capecitabine-resistant CRC patients after capecitabine adjuvant therapy. The expression level of CD16 was closely related to poor prognosis after capecitabine therapy. Importantly, the down-regulation of CD16 on CD11b^+^myeloid cells appeared as early as 1 month after capecitabine therapy in patients who were diagnosed with capecitabine-resistance by CT scans about 2–3 years after the treatment. The cut-off value of CD16 expression (3.8%) would be helpful for the prediction of capecitabine chemoresistance. Further analysis demonstrated that these CD11b^+^CD16^low/−^myeloid cells were mainly immature neutrophils and expression level of CD16 on neutrophils had a positive relationship with frequencies of anti-tumor immune cell populations, such as CD8^+^T cells and NK cells.

## Results

### CD16 expression levels on CD11b^+^myeloid cells in peripheral blood of capecitabine-resistant CRC patients are different from capecitabine-sensitive CRC patients after capecitabine therapy

**Fig. 1:**
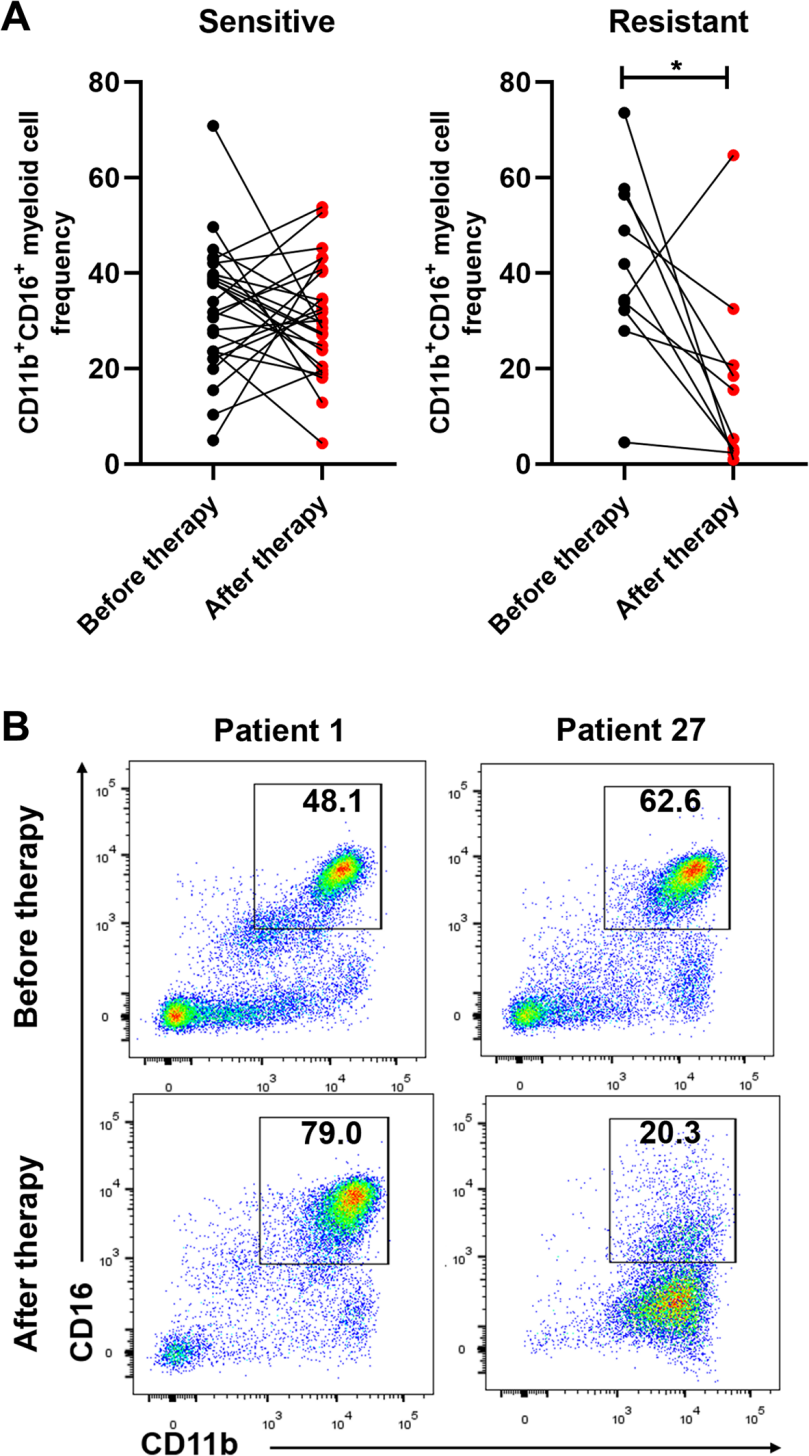
CD16 expression of peripheral blood myeloid cells were differential in CRC patients after capecitabine therapy. Peripheral venous blood from CRC patients received single-agent oral capecitabine adjuvant therapy was collected before the therapy and 6–9 months after the therapy and analyzed for myeloid cell-related markers. (Attention: Blood were collected 6–9 months after capecitabine treatment unless particularly noted). **a** Frequencies of CD11b^+^CD16^+^myeloid cells were compared before and after capecitabine therapy in capecitabine-sensitive and capecitabine-resistant patients (*n* = 26 in sensitive group and *n* = 10 in resistant group, respectively). **b** Representative images of CD16 expression on CD11b^+^myeloid cells before and after capecitabine therapy in two CRC patients from capecitabine-sensitive group or capecitabine resistant group, respectively. Diagnosis of drug-resistance was proved by CT scan during the follow-up in Fig. S1e. Mean ± SEM, *P<0.05 by t tests (**a**)

To explore if myeloid cells in peripheral blood could predict the treatment efficacy of capecitabine, we chose 36 CRC patients with capecitabine adjuvant treatment whose immune cells populations in peripheral blood were examined by flow cytometry before and about 6–9 months after the treatment. Patients were divided into capecitabine-sensitive and capecitabine-resistant groups, based on the diagnosis of recurrence by CT scan in about 2–3 years after capecitabine treatment (Table 1, Additional file 1: Fig. S1E). No significant change was observed in major myeloid cell subsets, such as monocytes (CD11b^+^CD14^+^CD15^−^), neutrophils (CD11b^+^CD15^+^CD14^−^ or CD11b^+^CD66b^+^CD14^−^) and MDSCs (CD11b^+^HLA-DR^-\low^CD33^+^), between capecitabine-sensitive patients and capecitabine-resistant patients (Additional file 1: S1A, B, C and D). But we found that the frequency of CD11b^+^CD16^+^myeloid cells was decreased in capecitabine-resistant patients after capecitabine treatment compared to that before the treatment (Fig. 1a). What’s important, a dramatic lower expression level of CD16 on CD11b^+^myeloid cells was observed in capecitabine-resistant patients, compared to that of drug-sensitive patients. Patient 1 and patient 27 are representative patients from capecitabine-sensitive group and capecitabine-resistant group, respectively (Fig. 1b). The diagnosis of capecitabine resistance was determined by CT scan (Additional file 1: Fig. S1E). However, when we analyzed these CD11b^+^CD16^+^myeloid cells in healthy donors (HDs) and CRC patients before capecitabine therapy, we found no difference between these two cohorts (Additional file 1: Fig. S1F and G). This indicated that change of CD16 expression on CD11b^+^CD16^+^myeloid cells was particular in CRC patients who were resistant to capecitabine therapy.

**Table 1:** Baseline characteristics of CRC patients in Fig. 1

Group	Number of Patients	Age	Sex	TNM Stage	Location	CEA (ng/ml)	CA199 (ng/ml)	Diagnosis of Recurrence After Capecitabine Treatment
Capecitabine-sensitive	1	40	M	II	Rectum	1.9	0.6	No
	2	62	M	II	Rectum	7.83	12.44	No
	3	56	M	II	Colon	1.71	24.79	No
	4	67	M	II	Colon	1.56	0.24	No
	5	81	M	II	Rectum	4.06	19.04	No
	6	32	F	II	Rectum	4	4.02	No
	7	50	F	II	Rectum	3.31	15.57	No
	8	62	F	II	Colon	2.41	3.57	No
	9	52	M	II	Rectum	4.3	19.58	No
	10	44	F	II	Colon	1.59	11.67	No
	11	72	M	II	Colon	4.44	17.52	No
	12	21	F	II	Colon	1.25	6.48	No
	13	53	M	II	Rectum	1.06	20.42	No
	14	63	M	III	Colon	0.8	16.51	No
	15	43	M	II	Rectum	1.08	0.9	No
	16	55	F	II	Rectum	1.18	4	No
	17	46	M	II	Rectum	4.92	18.26	No
	18	69	M	II	Rectum	0.89	23	No
	19	70	F	II	Colon	4.28	8.01	No
	20	71	F	II	Colon	4.62	20.58	No
	21	64	F	II	Rectum	2.2	19.31	No
	22	72	M	II	Rectum	1.19	2.28	No
	23	61	F	II	Rectum	8.81	4.4	No
	24	62	M	II	Rectum	1.92	28	No
	25	50	F	III	Colon	3.65	16.42	No
	26	65	M	III	Rectum	3.72	15.39	No
Capecitabine-resistant	27	63	M	II	Rectum	3.16	12.47	Yes
	28	70	M	III	Colon	5.28	11.1	Yes
	29	57	M	III	Colon	14.8	31.2	Yes
	30	65	F	II	Rectum	3.65	29.4	Yes
	31	52	M	II	Rectum	4.3	30.9	Yes
	32	49	M	III	Rectum	25.8	36.8	Yes
	33	54	M	III	Rectum	16.2	36.16	Yes
	34	54	F	III	Rectum	29.74	53.2	Yes
	35	45	F	II	Rectum	14	6.7	Yes
	36	59	M	II	Colon	4.37	12.4	Yes

### Decreased CD16 expression is correlated with poor pathological features in CRC patients after capecitabine therapy

To determine whether the expression level of CD16 on CD11b^+^ myeloid cells is related to treatment efficacy of capecitabine, we collected peripheral venous blood of 134 CRC patients 6–9 months after capecitabine treatment and divided these patients into two groups, CD16^+^ group and CD16^low/−^ group. Firstly, K-mean clustering algorithm was used to determine the boundary value to divide CD11b^+^CD16^+^myeloid cells into CD11b^+^CD16^high^cells and CD11b^+^CD16^low^cells, based on mean fluorescent intensity (MFI) of CD16 on CD11b^+^CD16^+^myeloid cells in peripheral blood after capecitabine therapy (Additional file 2: Fig. S2A). The boundary value of CD16 MFI for division of CD11b^+^CD16^high^ cells and CD11b^+^CD16^low^ cells was 7.1 × 10^3^. Next, we analyzed frequency of CD11b^+^CD16^high^ cells in peripheral blood after capecitabine therapy (Additional file 2: Fig. S2B), and determined the cut-off value for CD16 expression on CD11b^+^myeloid cells by receiver operating characteristic (ROC) analysis and Youden Index values (Additional file 2: Fig. S2C and S2D). The cut-off value was 3.8%. Patients of CD16^+^ group or CD16^low^ group were determined if their frequencies of CD11b^+^CD16^high^cells were higher or lower than the cut-off value (Additional file 2: Fig. S2B, S2C and S2D). Then we assessed correlations between the expression level of CD16 and CRC clinicopathological characteristics by χ^2^ test. The data revealed that patients in CD16^low/−^ group had more cancer recurrence (*P* = 0.042) and high level of carcinoembryonic antigen (CEA) (*P* = 0.023) as well as carbohydrate antigen 199 (CA199) (*P* = 0.016) compared to patients in CD16^+^ group (Table 2). There were 18 CRC patients (78.26%) developing recurrent tumor in CD16^low/−^ group, whereas only 5 cases (21.74%) were observed in CD16^+^ group. Among 20 CRC patients with high CEA level, 15 patients (75%) belonged to CD16^low/−^ group, while only 5 patients (25%) were CD16^+^. And 14 patients (77.78%) with high CA199 level were found in CD16^low/−^ group compared with 4 cases (22.22%) in that of CD16^+^. However, no significant difference was observed between these two groups on age, gender, tumor location, tumor size and Tumor Node Metastasis (TNM) stage (Table 2).

**Table 2:** Relationship between CD16 expression on CD11b^+^myeloid cells after capecitabine therapy and clinicopathologic characteristics

Characteristics	All patients (***n*** = 134)		CD16^**+**^ after therapy (***n*** = 65, 48.51%)		CD16^**low/−**^ after therapy (***n*** = 69, 51.49%)		***P*** value
	n	%	n	%	n	%	
Age (years)							
<65	70	52.24%	36	51.43%	34	48.57%	0.479
≥65	64	47.76%	29	45.31%	35	54.69%	
Gender							
Male	82	61.19%	42	51.22%	40	48.78%	0.43
Female	52	38.81%	23	44.23%	29	55.77%	
Tumor location							
Rectum	68	50.75%	32	47.06%	36	52.94%	0.733
Colon	66	49.25%	33	50.00%	33	50.00%	
Tumor Size							
≥5 cm	57	42.54%	25	43.86%	32	56.14%	0.354
<5 cm	77	57.46%	40	51.95%	37	48.05%	
CEA level after therapy							
≤5 ng/ml	114	85.07%	60	52.63%	54	47.37%	0.023
>5 ng/ml	20	14.93%	5	25.00%	15	75.00%	
CA199 level after therapy							
≤27 ng/ml	116	86.57%	61	52.59%	55	47.41%	0.016
>27 ng/ml	18	13.43%	4	22.22%	14	77.78%	
TNM stage (AJCC)							
Stage II	98	73.13%	45	45.92%	53	54.08%	0.322
Stage III	36	26.87%	20	55.56%	16	44.44%	
Location of recurrence							
Loco-regional	3	13.04%	2	40.00%	1	5.56%	0.042
liver + lung	3	13.04%	2	40.00%	1	5.56%	
liver	10	43.48%	1	20.00%	9	50.00%	
lung	6	26.09%	0	0.00%	6	33.33%	
peritoneum	1	4.35%	0	0.00%	1	5.56%	

**Fig. 2:**
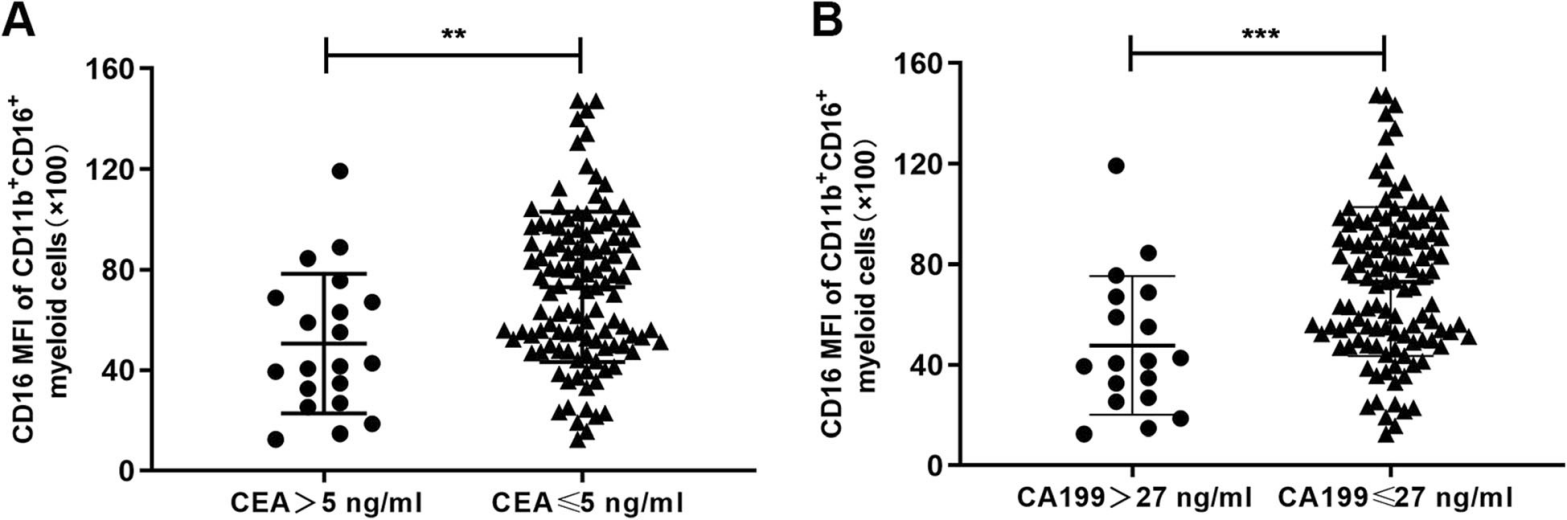
CD16 expression of CD11b^+^CD16^+^myeloid cells related to pathological features of CRC patients with capecitabine therapy. CRC patients receiving capecitabine therapy were divided into different groups according to their CEA or CA199 level (*n* = 20 in CEA-high (CEA > 5 ng) group and *n* = 114 in CEA-low (CEA ≤ 5 ng) group, *n* = 18 in CA199-high (CA199 > 27 ng) group and *n* = 116 in CA199-low (CA199 ≤ 27 ng) group). CD16 MFI of CD11b^+^CD16^+^myeloid cells in CRC patients acquired from flow cytometry analysis was compared between different groups. Mean ± SEM, **P<0.01, ***P<0.001 by t tests (**a, b**)

To further confirm these results, we divided 134 CRC patients after capecitabine treatment into two groups based on the level of CEA or CA199, and compared the expression level of CD16 on CD11b^+^CD16^+^myeloid cells between CEA-high (CEA > 5 ng) and CEA-low (CEA ≤ 5 ng) groups, or between CA199-high (CA199 > 27 ng) and CA199-low (CA199 ≤ 27 ng) groups. The boundary value of CEA and CA199 were decided by clinical guidelines. The results showed that the expression level of CD16 was dramatically decreased in either CEA-high or CA199-high groups compared to CEA-low or CA199-low groups (Fig. 2a and b), suggesting that the decreased expression level of CD16 on CD11b^+^myeloid cells after capecitabine treatment was related to the poor pathological features. In conclusion, low level of CD16 expression was related to poor pathological features, such as tumor recurrence, CEA and CA199, in CRC patients with capecitabine therapy.

### CD16 serves as a prognostic marker for CRC patients received capecitabine adjuvant chemotherapy

**Fig. 3:**
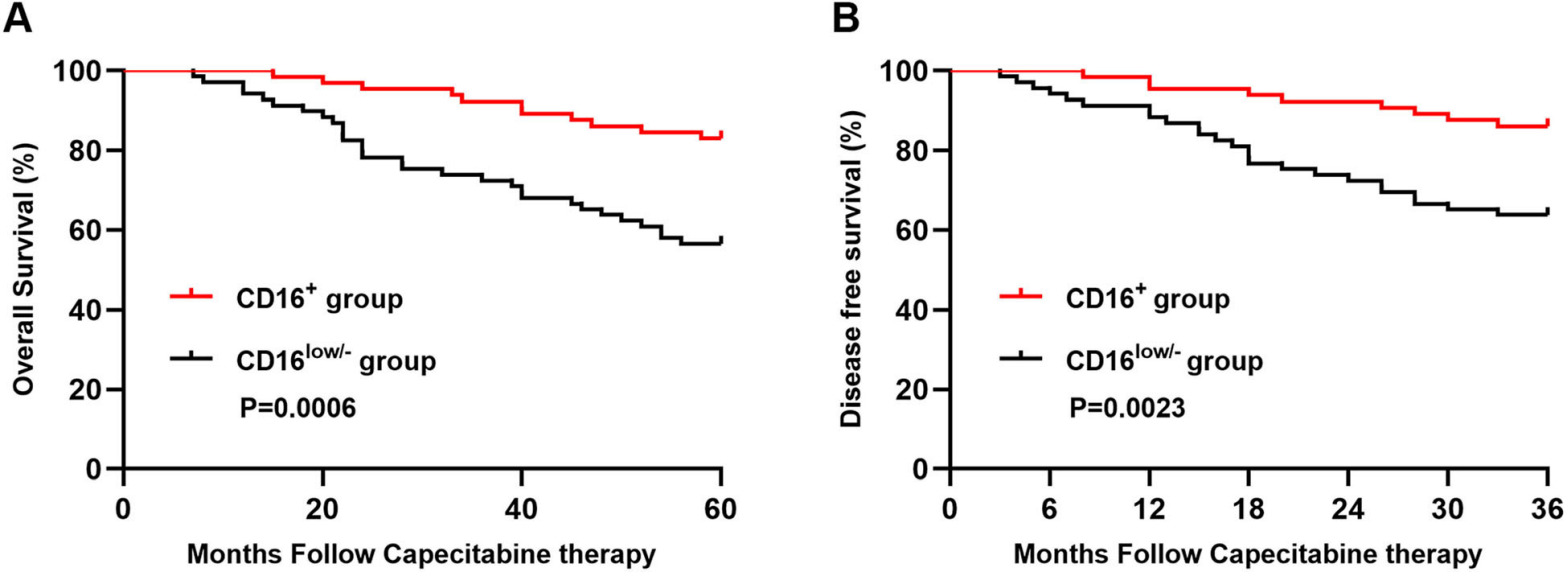
CD16 high expression on CD11b^+^myeloid cells was good prognostic marker for CRC patients’ survival. Kaplan-Meier analysis of overall survival (OS) and disease free survival (DFS) was performed in CD16^+^ group and CD16^low/−^ group, *p* values were calculated by log-rank test (*n* = 65 in CD16^+^ group and *n* = 69 in CD16^low/−^ group)

To further explore the prognostic significance of CD16 expression on CD11b^+^myeloid cells in predicting the treatment efficacy of capecitabine chemotherapy, we compared the differences of overall survival (OS) and disease free survival (DFS) between CD16^+^ group and CD16^low/−^ group. The survival curves revealed that there were significant association between the expression level of CD16 and OS (*P* = 0.0006)(Fig. 3a) or DFS (*P* = 0.0023)(Fig. 3b), suggesting that low expression level of CD16 was associated with shorter survival. Next, we used univariate analysis to further elucidate the significance of CD16 expression in predicting prognosis of CRC patients receiving capecitabine. The result demonstrated that CD16 expression level (*P* = 0.011, HR = 0.395) was prognostic factor for OS (Table 3). What’s important, Cox multivariate analysis also demonstrated that expression level of CD16 (*P* = 0.049, HR = 2.13) was still independent predictors of OS (Table 3). These results demonstrated that the expression level of CD16 on CD11b^+^myeloid cells may serve as a good prognostic marker for overall survival in CRC patients with capecitabine adjuvant chemotherapy.

**Table 3:** Univariate and multivariate analyses for survival in CRC patients after capecitabine therapy

Prognostic parameter	Univariate analysis			Multivariate analysis		
	HR	95%CI	p value	HR	95%CI	***p*** value
CD16 expression	0.395	0.194–0.808	0.011	2.130	0.227–0.998	0.049
Gender	1.049	0.534–2.064	0.889	–	–	–
Age	0.768	0.393–1.501	0.441	–	–	–
Tumor location	0.911	0.469–1.768	0.783	–	–	–
Tumor size	1.102	0.567–2.144	0.774	–	–	–
CEA	2.410	1.093–5.313	0.029	1.868	0.831–4.201	0.131
CA199	1.624	0.674–3.914	0.280	–	–	–
TNM	1.443	0.707–2.947	0.314	–	–	–
Recurrence	2.516	1.207–5.243	0.014	2.130	1.012–4.484	0.046

*HR* Hazard ratio, *CI* Confident interval

### Down-regulation of CD16 expression on CD11b^+^myeloid cells appears earlier than diagnosis of capecitabine by imaging tests

**Fig. 4:**
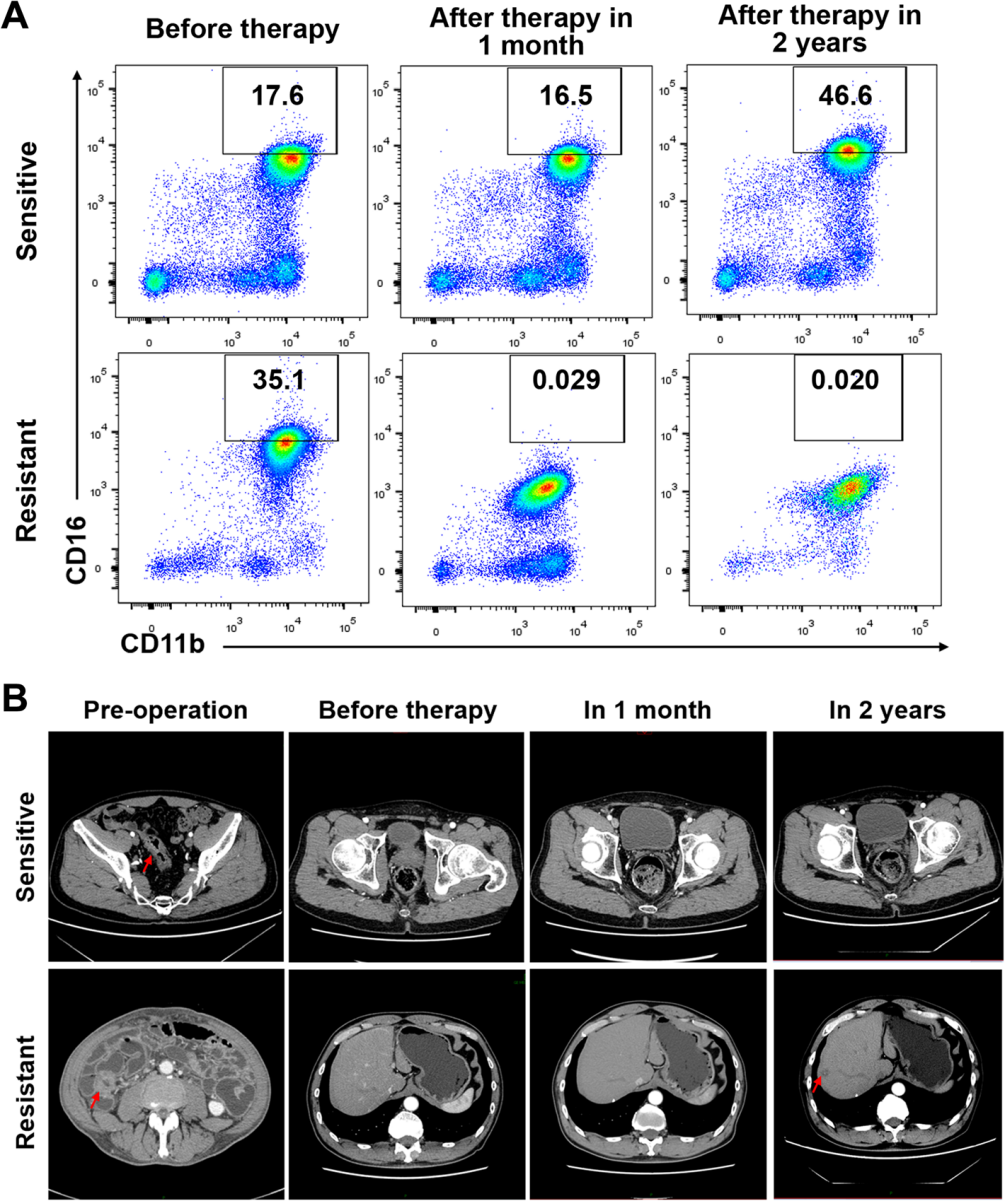
Analysis of CD16 expression was more sensitive than CT scan after capecitabine therapy. **a** Peripheral venous blood from CRC patients receiving single-agent oral capecitabine adjuvant therapy was collected at different time (before capecitabine therapy, 1 month and 2 years after the therapy). Frequencies of CD11b^+^CD16^high^myeloid cells were analyzed by flow cytometry. **b** CT scan was performed during follow-up after adjuvant chemotherapy in same patients as that of (**a**) respectively. Sensitive patient, normal operation site with no recurrence. Resistant patient, resectable metachronous liver metastases (red arrows)

As we know, adjuvant chemotherapy remains the first line therapy for CRC patients. Capecitabine, the oral prodrug of 5-fluorouracil, is one of the primary drugs for the treatment. A number of CRC patients become insensitive to the therapy and suffer from cancer recurrence. In clinic, capecitabine-resistance is mainly diagnosed by cancer recurrence discovered through colonoscopy or CT scan in about 2–3 years after capecitabine treatment [18]. Next, we wondered if the change of CD16 expression level on CD11b^+^myeloid cells appeared earlier than CT-showed recurrence. We selected CRC patients with capecitabine treatment whose blood samples were examined before and after capecitabine treatment (Table 1). The results showed in 90% patients in capecitabine-resistant group, the frequency of CD11b^+^CD16^+^myeloid cells was decreased 6–9 months after treatment compared to that before treatment (Fig. 1a), while capecitabine resistance was diagnosed by CT scan about 2 years after the treatment (Table 1 and Additional file 1: Fig. S1E). What’s important, in a resistant patient, decreased expression level of CD16 was found as early as 1 month after capecitabine treatment (Fig. 4a). The frequency of CD11b^+^CD16^high^ cell population was largely lower than the cut-off value (3.8%). Nevertheless, 15 months after the capecitabine therapy, tumor recurrence was found in the liver from CT scan (Fig. 4b). These data suggested that down-regulation of CD16 on CD11b^+^myeloid cells served as a more sensitive examine than CT in CRC patients treated with capecitabine.

### CD11b^+^CD16^low/−^myeloid cells are mainly immature neutrophils after capecitabine therapy

**Fig. 5:**
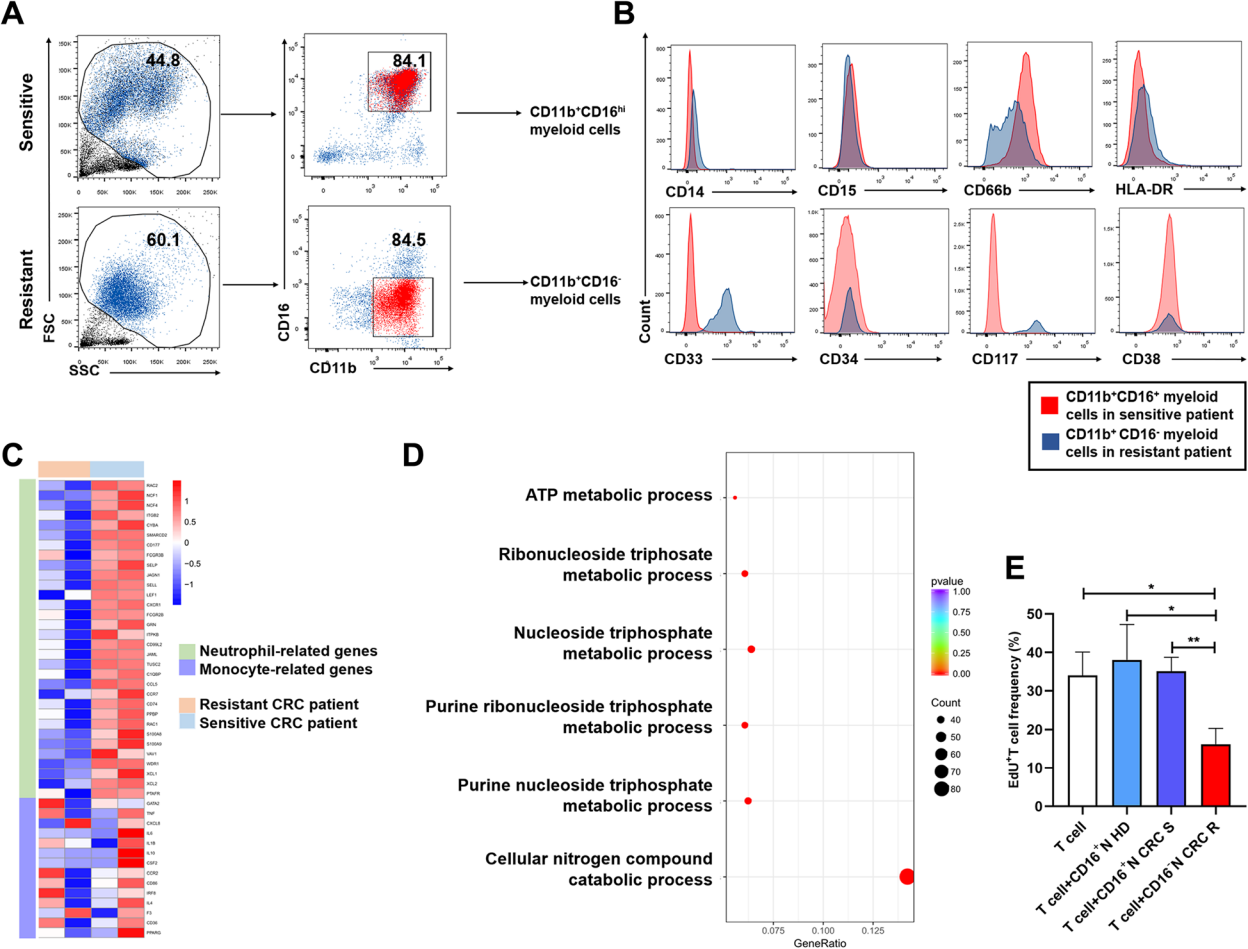
CD11b^+^CD16^+^myeloid cells became immature neutrophils after therapy in capecitabine-resistant patients. **a** Peripheral venous blood from capecitabine-resistant and capecitabine-sensitive CRC patients was collected after the treatment in 6–9 months. CD11b^+^CD16^+^myeloid cells in sensitive patients and that of CD11b^+^CD16^−^ in resistant patients were sorted for further analysis in (**b**), (**c**) and (**d**). **b** Expression of myeloid-associated and hematopoietic progenitor-associated markers on CD11b^+^CD16^+^myeloid cells in sensitive patients and on CD11b^+^CD16^−^myeloid cells in resistant patients was analyzed by flow cytometry. **c** Peripheral blood CD11b^+^CD16^+^myeloid cells in sensitive patients and CD11b^+^CD16^−^myeloid cells in resistant patients were sorted and analyzed by RNA sequencing. Expression of neutrophil-related and monocyte-related genes derived from the results of RNA sequencing was shown in the heatmap. **d** GO enrichment terms of differentially expressed MDSC-related immunosuppressive biological processes derived from RNA sequencing. **e** Autologous T cells were cultured alone, cocultured with peripheral blood CD11b^+^CD16^+^myeloid cells (from HDs and sensitive CRC patients) or CD11b^+^CD16^−^myeloid cells (from resistant CRC patients) for 48 h, respectively. Proliferation of T cells were analyzed by flow cytometry after incubation (*n* = 3 for each group). CD16^+^N HD = CD11b^+^CD16^+^myeloid cells from HDs, CD16^+^N CRC S = CD11b^+^CD16^+^myeloid cells from sensitive CRC patients, CD16^−^N CRC R = CD11b^+^CD16^−^myeloid cells from resistant CRC patients. Mean ± SEM, *P<0.05, **P<0.01 by t tests (**e**)

To further characterize the population of CD11b^+^CD16^low/−^myeloid cells, we isolated CD11b^+^CD16^+^myeloid cells from capecitabine-sensitive patients and CD11b^+^CD16^−^myeloid cells from capecitabine-resistant patients after capecitabine therapy (Fig. 5a). The data from flow cytometry revealed that these two populations were mainly neutrophils proved by their CD15 and CD66b expression (Additional file 3: Fig. S3A). To further verify these CD11b^+^CD16^−^myeloid cells and CD11b^+^CD16^+^myeloid cells were both neutrophils, we sorted these cells from capecitabine-resistant patients and capecitabine-sensitive patients, respectively. Characteristics of these patients were listed in Additional file 4: Table S1. We compared our data of RNA sequencing with published data of neutrophils from Jiang K et al. [30] using gene set enrichment analysis (GSEA). The results revealed that, in gene sets of neutrophil signature, the expression pattern of these cells was similar to that of the neutrophils provided by other group (Additional file 3: Fig. S3B, Additional file 5: Table S2). Nevertheless, the decline of CD15 and CD66b expression, combine with the elevation of hematopoietic progenitor-related markers, especially CD33 and CD117, suggested that these CD11b^+^CD16^−^myeloid cells in capecitabine-resistant patients became more immature after the therapy compared with CD11b^+^CD16^+^myeloid cells from capecitabine-sensitive patients (Fig. 5b). The data of RNA sequencing also revealed declined expression of some neutrophil-related genes in CD11b^+^CD16^−^myeloid cells from capecitabine-resistant patients after capecitabine therapy, which implied immature status of these neutrophils (Fig. 5c). In addition, active metabolism of nitrogen species, purine nucleoside and ATP were also found in these CD11b^+^CD16^−^myeloid cells, which are tightly related to immunosuppressive role of MDSC [24, 30] (Fig. 5d). To verify the immunosuppressive role of these CD11b^+^CD16^−^myeloid cells, we sorted peripheral blood CD11b^+^CD16^−^myeloid cells from capecitabine-resistant CRC patients, and CD11b^+^CD16^+^myeloid cells from capecitabine-sensitive CRC patients or HDs, and autologous T cells as well. After coculture T cells with these myeloid cells in the presence of leukocyte activators, proliferation of T cell was significantly declined in resistant CRC patients group, compared with single T cell group, HD group and sensitive CRC patients group (Fig. 5e). The results suggested that these CD11b^+^CD16^−^myeloid cells in capecitabine-resistant patients might exert immature cell status and play immunosuppressive role like MDSC.

### The low expression level of CD16 on neutrophils is related to pro-tumor status in CRC patients after capecitabine therapy

**Fig. 6:**
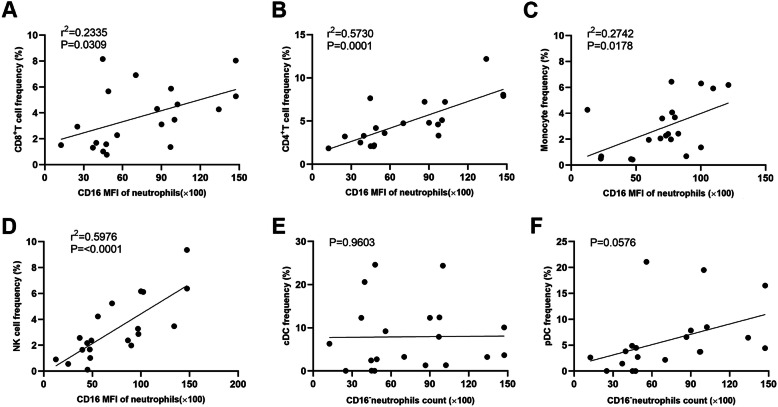
CD16 low expression on neutrophils predicted pro-tumor immune status in CRC patients with capecitabine therapy. Peripheral venous blood from CRC patients received single-agent oral capecitabine adjuvant therapy was collected 6–9 months after the therapy and analyzed for different immune cell subsets by flow cytometry. CD16 MFI of peripheral blood neutrophils was calculated by flow cytometry analysis, and the correlations between CD16 MFI of neutrophils and frequencies of CD8^+^ T cells (**a**), CD4^+^ T cells (**b**), monocytes (**c**), NK cells (**d**), cDCs (**e**) and pDCs (**f**) among total peripheral blood leukocytes were analyzed by Pearson’s correlation test

As we know, immature myeloid cells are usually MDSCs, which could exert powerful immunosuppressive role, especially in inhibiting T cells and NK cells [24, 25, 30]. As our results showed that CD11b^+^CD16^+^myeloid cells from capecitabine-sensitive patients and CD11b^+^CD16^−^myeloid cells from capecitabine-resistant patients were mainly neutrophils, we tried to find out the relationship between the expression level of CD16 on neutrophils and other major immune cell subsets. We collected peripheral venous blood from colorectal cancer patients 6–9 months after capecitabine therapy and analyzed frequencies of immune cells by flow cytometry. The relationships between expression level of CD16 on neutrophils and frequencies of immune cell subsets were analyzed by Pearson’s correlation test. The results showed that CD16 expression was positively related to CD8^+^T cell, CD4^+^T cell, monocyte and NK cell frequencies (Fig. 6a, b, c and d), but not that of cDC and pDC in patients after capecitabine therapy (Fig. 6e and f), suggesting that CD16^low/−^neutrophils might have immunosuppressive activity as MDSCs.

## Discussion

Over the past few decades, numerous researchers have attempted to improve the efficacy of capecitabine adjuvant therapy to ameliorate prognosis of CRC patients. However, it remains one of the principal obstacle for cancer therapy at present. In this study, we demonstrated that the expression level of CD16 was down-regulated in capecitabine-resistant patients and lower expression level of CD16 on neutrophils in peripheral blood was correlated with poor prognosis in CRC patients with capecitabine adjuvant therapy. Importantly, down-regulation of CD16 was observed as early as 1 month after capecitabine treatment, which was more sensitive than CT scan, indicating its great value in clinical application. We determined the cut-off value of CD16 expression (3.8%) on neutrophils for the prediction of capecitabine chemoresistance, which would be helpful for clinical application and further researches. Analyzation of these CD16^low/−^neutrophils in capecitabine-resistant patients revealed their immature status, and the expression of CD16 on neutrophils was positively correlated with frequencies of anti-tumor immune cell populations.

To this day, coloscopy and CT scan are still the main examines to supervise CRC progression and discover recurrence, which is vital for capecitabine-resistance diagnosis. Unfortunately, these two methods could only provide evidence until tumors are big enough to be discovered, patients won’t have enough time to adjust the treatment. CEA and CA199 are widely used to CRC surveillance as well, especially CEA [31]. However, CEA and CA199 cannot predict cancer progression so precisely, and the false positive or negative results will lead to anxiety and excessive therapy. What’s more, some clinical trial also suggested that combining CEA and CT got no advantage compared with single examine [32]. In this study, our results showed that CD16 expression could serve as a good prognostic marker for poor CRC progression after capecitabine therapy. Analyzation of CD16 expression has great advantages. First, the down-regulation of CD16 expression on neutrophils could be observed at the early stage of capecitabine-resistance after the treatment (Fig. 4). Previous studies have demonstrated that 85% CRC patients had primary resistance to 5-FU single treatment [12, 13], thus the marker is essential for the drug-selection in these patients. Second, this marker is quite accurate for predicting capecitabine-resistance after the therapy. In our study, we collected totally 134 CRC patients with capecitabine therapy to examine the expression level of CD16 on neutrophils. Among 23 patients who were diagnosed as capecitabine-resistance, 78% patients were observed to have down-regulation of CD16 in 6–9 months after capecitabine treatment (Table 2). Third, the examination of CD16 expression only takes about 3 ml peripheral blood, and it is noninvasive and has nearly no effect on patients’ health.

Capecitabine, the oral form of 5-FU, which is widely used in CRC therapy, has only modest efficacy due to the chemoresistance. Great efforts have been taken to find out the mechanism. Previous studies mainly concentrated on tumor cells themselves, such as expression of specific genes or generation of particular tumor cells [33, 34]. In this research, we worked on the correlation between changes on immune system and capecitabine chemoresistance, and illustrated the conversion from neutrophils to immunosuppressive, PMN-MDSC-like neutrophils in these capecitabine insensitive patients by RNA sequencing and flow cytometry. Our conclusion could also be supported by other studies, that 5-FU could promote MDSC pro-tumor function. The study by Bruchard M et al. found that 5-FU could activate NLRP3 inflammasome in MDSC and promote tumor growth [27]. Gobbo J et al. also discovered that 5-FU facilitated production of tumor-derived HSP70 exosomes, which favored MDSC activation [28]. Thus, prevention of MDSC function after capecitabine, or 5-FU therapy holds great promise for improving drug efficacy.

Researchers have revealed that CD16^+^myeloid cells were tightly related to CRC development [35, 36]. Giulio S et al. found that CD16^+^myeloid cell infiltration in CRC tumor tissue represented favorable prognosis [35], and by using in vitro studies, these studies also demonstrated that colon cancer infiltrate neutrophils enhance the responsiveness of CD8^+^ T cells by T-cell receptor triggering [36]. Our work differed from theirs in some ways. Firstly, our study focused on CRC patients who received capecitabine adjuvant treatment after surgery, while Giulio Spagnoli group focused on all CRC patients and some healthy donors. Secondly, biopsies from different positions were analyzed. Peripheral blood was used in our study, while Giulio Spagnoli group mainly focused on tumor biopsies. Except these differences, some of our results were also consistent with studies from Giulio Spagnoli group. Firstly, both our data and Giulio Spagnoli group’s data found that phenotype of peripheral blood CD11b^+^CD16^+^myeloid cells had no difference between healthy donors and CRC patients without capecitabine therapy (Fig. S1F and G). Secondly, our work indicated that CD16 high/positive expression after capecitabine therapy predicted sensitivity to the therapy and good prognosis. These results were consistent with the work from Giulio Spagnoli group, that CD16^+^myeloid cells related to good prognosis of CRC patients.

MDSCs are a heterogeneous population of myeloid cells stay at different stages of differentiation. PMN-MDSCs are a great part of MDSCs that could be considered as counterparts of immature granulocytes, chiefly immature neutrophils [23]. In this study, we found down-regulation of CD16 expression on myeloid cells in capecitabine-insensitive CRC patients after capecitabine treatment. These CD16^low/−^myeloid cells after the therapy were mainly immature neutrophils. CD16 is a low affinity Fcγ receptor, which could activate antibody-dependent process like phagocytosis in neutrophils and other phagocytes [37]. It is expressed on neutrophils during the maturation. Researchers also revealed that CD16 is typically associated with PMN activation and phagocytosis, and its expression will change in different maturation status [38, 39]. MDSCs could exert pro-tumor roles, mainly through inhibition of effective T cells and NK cells [24, 25]. Our study demonstrated that low expression of CD16 on neutrophils after the therapy was related to decreased frequencies of anti-tumor immune cells, like CD8^+^T cells and NK cells, suggesting that they may have immunosuppressive activity as MDSCs. The mechanism underlying the changes induced by capecitabine would be investigated further, and it could be a good target to compete against capecitabine chemoresistance.

## Conclusions

In conclusion, CD16 seems to be a promising target for CRC progression surveillance after capecitabine therapy. Studies of CD16 expression on neutrophils may light the path for not only predicting prognosis but also solving capecitabine resistance in CRC patients.

## Methods

### Patients and peripheral blood

Peripheral venous blood of CRC patients in Department of Gastrointestinal Surgery, Renji Hospital (Shanghai, China) from January 2012 to December 2018 was gotten before capecitabine adjuvant treatment and at different time after the treatment (as indicated in figure legend). Peripheral venous blood of healthy donors was gotten in Renji Hospital. The pathological information of 134 patients was retrieved from the Pathology Department of Renji Hospital. These peripheral blood was used for flow cytometric analysis. All the patients were provided with written informed consent before enrolment, and the study was approved by the Research Ethics Committee of Shanghai Jiao Tong University School of Medicine Renji Hospital (Approval No. Renji [2013] N013). None of patients had received radiotherapy or chemotherapy before surgery. All patients were followed-up until death or until the final follow-up (May 2019).

### Flow cytometry analysis

Peripheral venous blood samples were subjected to density centrifugation using Ficoll-hypaque solution to isolate mononuclear cells and granulocytes. Cell suspension was treated with BD Pharm Lyse lysing solution for red blood cell lysing. To determine the frequency of different subsets myeloid cell subsets, these cells (10^6 cells/tube) were stained by monoclonal antibodies BB515 Rat anti-CD11b (BD Biosciences, Clone # M1/70), PE Mouse anti-human CD66b (BD Biosciences, Clone # G10F5) or PE mouse IgM κ isotype control (BD Biosciences, Clone # G155–228), BV650 mouse anti-human CD16 (BD Biosciences, Clone # 3G8), PerCP-Cy5.5 mouse anti-human CD15 (BD Biosciences, Clone # HI98) or PerCP-Cy5.5 mouse IgM κ isotype control (BD Biosciences, Clone # G155–228), PE-Cy7 mouse anti-human CD14 (BD Biosciences, Clone # M5E2) and BV421 mouse anti-human CD33 (BD Biosciences, Clone # WM53) at 4 °C for 30 min respectively. Flow cytometry was performed on an LSRFortessa (BD Biosciences) and analyzed using FlowJo Version X (Tree Star, Inc).

### Myeloid cells immunosuppressive activity assay

Peripheral blood CD11b^+^CD16^−^myeloid cells (from resistant CRC patients after capecitabine therapy), CD11b^+^CD16^+^myeloid cells (from HD and sensitive CRC patients after capecitabine therapy) and autologous T cells were sorted by FACS Aria II(BD Biosciences). T cells were either cultured alone, or cocultured with sorted myeloid cells at the ratio 2:1 (5 × 10^5^/ml, respectively) in RPMI 1640 medium with 10% FBS. Anti-CD3/CD28 stimulation beads (Invitrogen) and 100 u/ml IL-2 (Peprotech) were added to stimulate T cells. After 48 h, T cells were staining with 10 mM EdU (Abcam) and cultured for another 24 h. T cell proliferation was analyzed by flow cytometry.

### RNA sequencing

Peripheral venous blood samples was subjected to density centrifugation using Ficoll-hypaque solution to isolate mononuclear cells and granulocytes. After lysing RBCs, cells were incubated with BB515 Rat anti-CD11b (BD Biosciences) and BV650 mouse anti-human CD16 (BD Biosciences) antibodies and sorted by flow cytometry using BD FACS AriaII. RNA sequencing (RNA-seq) was performed on sorted CD11b^+^CD16^+^myeloid cells in capecitabine-sensitive patients and CD11b^+^CD16^−^myeloid cells in capecitabine-resistant patients. Qubit 2.0 (Life Technologies, USA) and Bioanalyzer 2100 (Agilent, Germany) were used to analyze the RNA quality and integrity. A total of 3 μg RNA was used for the RNA sample preparations. Extracted RNA samples were processed using the NEBNext® UltraTM RNA Library Prep Kit (Lexogen) and sequenced on an Illumina Hiseq X-Ten with control of Hiseq Control Software (HCS). The sequencing library were qualified by Qubit 2.0 (Life technologies, USA) and Bioanalyzer 2100 (Agilent, Germany). Raw reads were processed through in-house perlscripts. Clean reads were obtained by removing reads containing adapter. Differentially expressed genes were defined by *P* < 0.05 and an absolute fold change > 2. Using Gene Set Enrichment Analysis, enrichment of a specific gene set was tested, and core enrichment genes were determined.

### Statistical analysis

All analyses in this study were performed by IBM SPSS STATISTICS 22.0 software and GraphPad Prism 8 software. Statistical analysis of the data was performed using the Student’s t-test and χ^2^ test, and results were presented as mean ± SEM unless indicated. Unsupervised K-means clustering of CD11b^+^CD16^high^cells and CD11b^+^CD16^low^cells, Receiver operating characteristic (ROC) and Youden Index values were used to determine cut-off value of CD16 expression on CD11b^+^CD16^+^myeloid cells. Univariate and multivariate Cox regression analyses were used to identify independent prognostic factors. The results were considered statistically significant if *P* < 0.05.

## Supplementary information

**Additional file 1: Figure S1.** Flow cytometry analyses of different myeloid cell subsets and representative CT scan in CRC patients. Peripheral venous blood from CRC patients received single-agent oral capecitabine adjuvant therapy was collected 6–9 months after the therapy and analyzed for myeloid cell-related markers. Frequencies of different myeloid cell subsets, including monocytes (CD11b^+^CD14^+^CD15^−^)(A), neutrophils (CD11b^+^CD15^+^CD14^−^or CD11b^+^CD66b^+^CD14^−^) (B and C) and MDSC(CD11b^+^ HLA-DR^-\low^CD33^+^)(D) were compared between capecitabine-sensitive patients and capecitabine-resistant patients (*n* = 26 in capecitabine-sensitive group and *n* = 10 in capecitabine-resistant group, respectively). (E) CT scan was performed before the operation and during follow-up in same patients as that of Fig. 1b. Patient 1, normal operation site and no recurrence. Patient 27, unresectable metachronous lung metastases. Red arrows indicate primary tumor in situ or metastatic sites. (F) Peripheral blood CD11b^+^CD16^+^ myeloid cells from HDs and CRC patients before therapy were analyzed using flow cytometry. (G) CD66b, CD15, CD14, CD34 and CD117 expression on CD11b^+^CD16^+^ myeloid cells in (F) were analyzed by flow cytometry.

**Additional file 2: Figure S2**. CRC patients were divided into CD16^+^group and CD16^low^group based on CD16 MFI of CD11b^+^myeloid cells. (A) Unsupervised K-means clustering of CD11b^+^CD16^high^cells and CD11b^+^CD16^low^cells based on CD16 MFI of CD11b^+^CD16^+^myeloid cells in peripheral blood (K = 2) after capecitabine therapy. CD11b^+^CD16^high^cells (above red dashed line, *n* = 65). (B) Peripheral venous blood was taken from 134 CRC patients 6–9 months after capecitabine therapy and frequencies of CD11b^+^CD16^high^cells were analyzed by flow cytometry. (C) Receiver operating characteristic (ROC) was used for determination of different cut-off values for CD16 expression level of CD11b^+^myeloid cells based on frequencies of CD11b^+^CD16^high^cells in peripheral blood of CRC patients after capecitabine therapy. (D) Youden Index values were calculated for different cut-off values of ROC curve. Dashed line indicated the empirically chosen cut-off value for CD16 expression level of CD11b^+^myeloid cells (3.8%). Patients of CD16^+^ group or CD16^low^ group were determined if their frequencies of CD11b^+^CD16^high^ cells were higher or lower than the cut-off value.

**Additional file 3: Figure S3.** CD11b^+^CD16^+^ myeloid cells and CD11b^+^CD16^−^ myeloid cells were neutrophils. (A) Peripheral venous blood from capecitabine-resistant and capecitabine-sensitive CRC patients after the therapy was collected. CD11b^+^CD16^+^ myeloid cells in sensitive patients and CD11b^+^CD16^−^ myeloid cells in resistant patients were analyzed for their CD15 and CD66b expression. Meanwhile, a part of peripheral blood leukocytes from same patients were stained with mouse IgM isotype control. CD15 and CD66b expression were compared between cells stained with antibodies and that stained with isotype control. (B) The data of RNA sequencing was compared with published data of neutrophils using GSEA. Representative gene sets of neutrophil signature were shown.

**Additional file 4: Table S1.** Baseline characteristics of CRC patients in RNA sequencing.

**Additional file 5: Table S2.** Gene set enrichment analysis of neutrophil signature. The data of RNA sequencing was compared with published data of neutrophils using GSEA.

## Data Availability

The majority of the data used to support the findings of this study are included in the article and additional files. The rest data are available from the corresponding author upon request.

## Abbreviations

CA: Carbohydrate antigen
CD: Cluster of differentiation
CEA: Carcinoembryonic antigen
CRC: Colorectal cancer
CT: Computed tomography
DFS: Disease free survival
GSEA: Gene set enrichment analysis
HD: Healthy donor
MDSC: Myeloid-derived suppressor cell
MFI: Mean fluorescent intensity
OS: Overall survival
ROC: Receiver operating characteristic
TNM: Tumor Node Metastasis
5-FU: 5-fluorouracil
