# Supplementary figures and images for: CD16 expression on neutrophils predicts treatment efficacy of capecitabine in colorectal cancer patients
Source: BMC Immunol. 2020 Aug 8;21:46. doi: 10.1186/s12865-020-00375-8 (PMC7414545; doi:10.1186/s12865-020-00375-8)

Fig S1

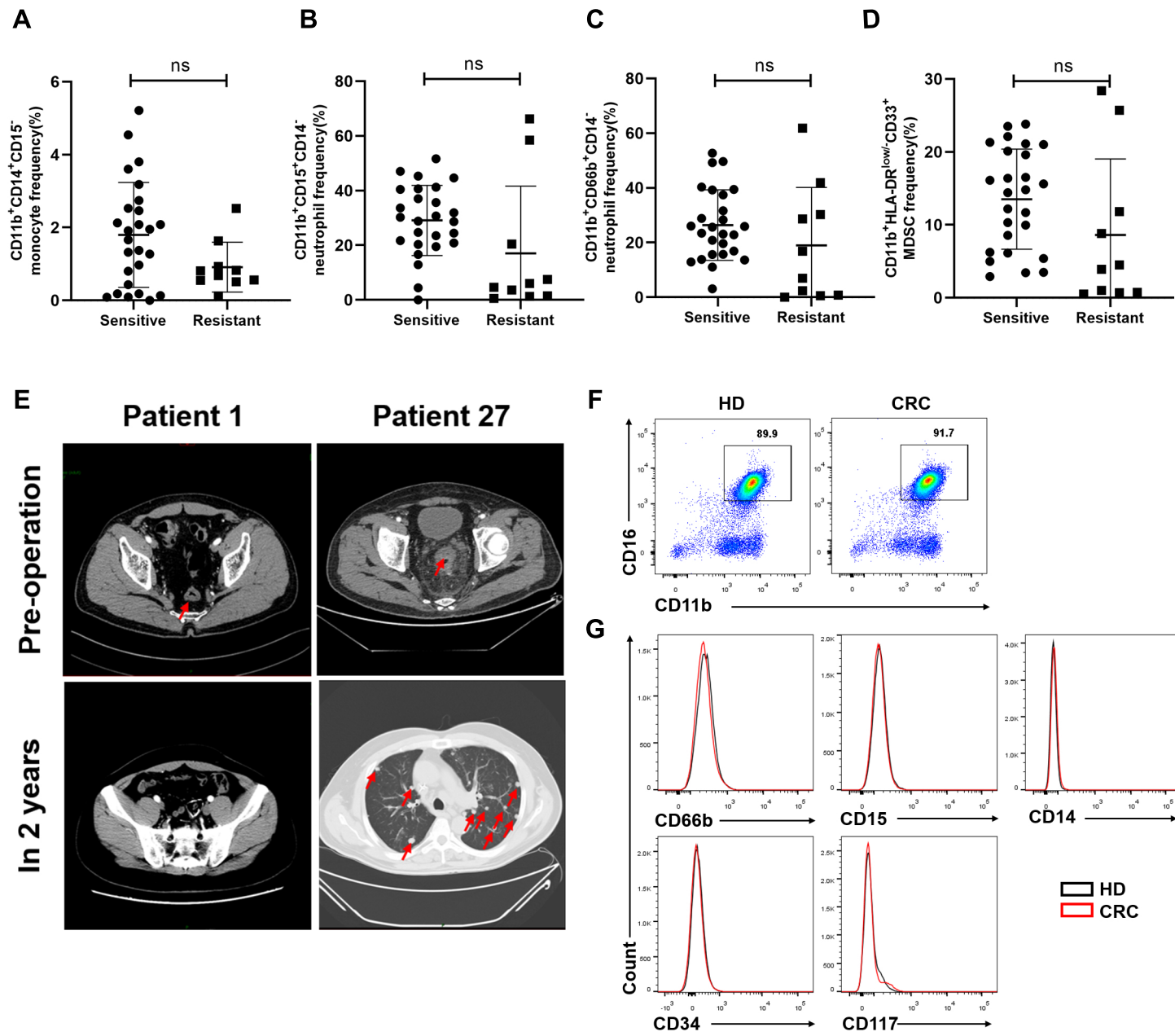

Supplement: Supplementary file 1 — Additional file 1: Figure S1. Flow cytometry analyses of different myeloid cell subsets and representative CT scan in CRC patients. Peripheral venous blood from CRC patients received single-agent oral capecitabine adjuvant therapy was collected 6–9 months after the therapy and analyzed for myeloid cell-related markers. Frequencies of different myeloid cell subsets, including monocytes (CD11b+CD14+CD15−)(A), neutrophils (CD11b+CD15+CD14−or CD11b+CD66b+CD14−) (B and C) and MDSC(CD11b+ HLA-DR-\lowCD33+)(D) were compared between capecitabine-sensitive patients and capecitabine-resistant patients (n = 26 in capecitabine-sensitive group and n = 10 in capecitabine-resistant group, respectively). (E) CT scan was performed before the operation and during follow-up in same patients as that of Fig. 1b. Patient 1, normal operation site and no recurrence. Patient 27, unresectable metachronous lung metastases. Red arrows indicate primary tumor in situ or metastatic sites. (F) Peripheral blood CD11b+CD16+ myeloid cells from HDs and CRC patients before therapy were analyzed using flow cytometry. (G) CD66b, CD15, CD14, CD34 and CD117 expression on CD11b+CD16+ myeloid cells in (F) were analyzed by flow cytometry. [file 12865_2020_375_MOESM1_ESM.pdf]

Fig S2

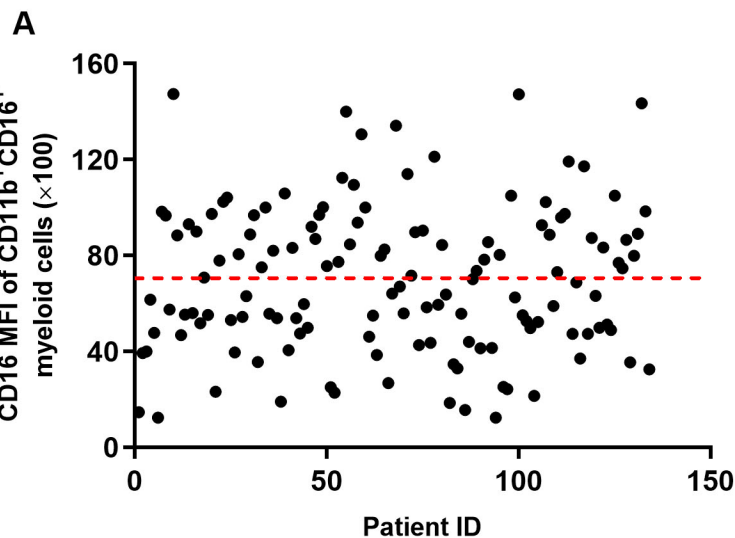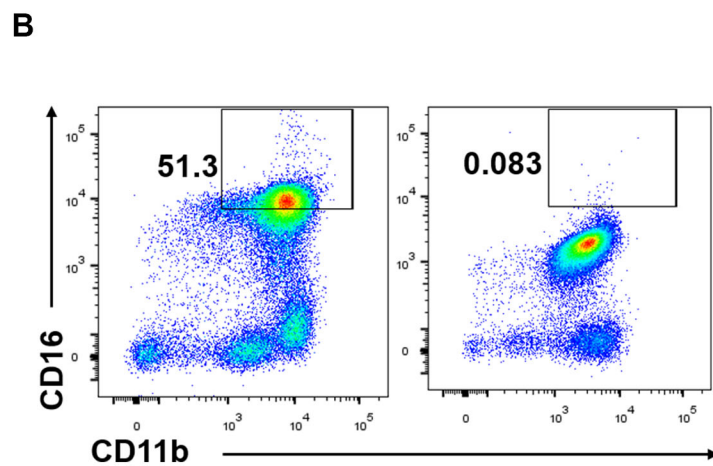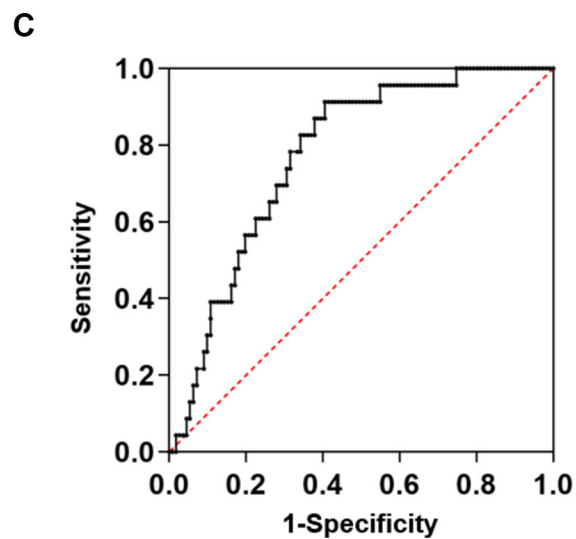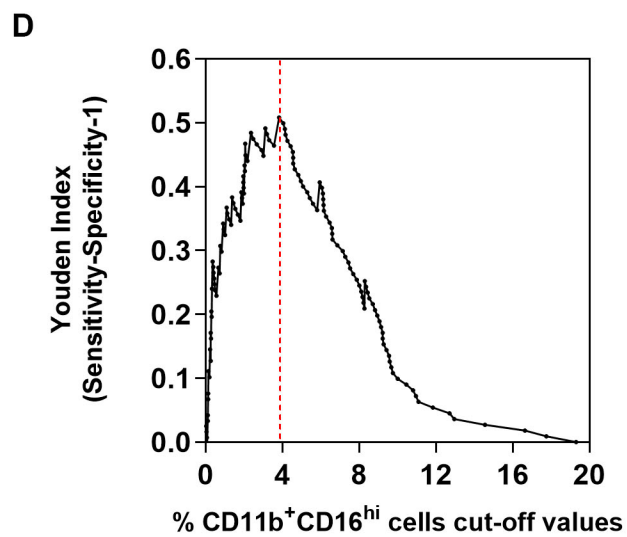

Supplement: Supplementary file 2 — Additional file 2: Figure S2. CRC patients were divided into CD16+group and CD16lowgroup based on CD16 MFI of CD11b+myeloid cells. (A) Unsupervised K-means clustering of CD11b+CD16highcells and CD11b+CD16lowcells based on CD16 MFI of CD11b+CD16+myeloid cells in peripheral blood (K = 2) after capecitabine therapy. CD11b+CD16highcells (above red dashed line, n = 65). (B) Peripheral venous blood was taken from 134 CRC patients 6–9 months after capecitabine therapy and frequencies of CD11b+CD16highcells were analyzed by flow cytometry. (C) Receiver operating characteristic (ROC) was used for determination of different cut-off values for CD16 expression level of CD11b+myeloid cells based on frequencies of CD11b+CD16highcells in peripheral blood of CRC patients after capecitabine therapy. (D) Youden Index values were calculated for different cut-off values of ROC curve. Dashed line indicated the empirically chosen cut-off value for CD16 expression level of CD11b+myeloid cells (3.8%). Patients of CD16+ group or CD16low group were determined if their frequencies of CD11b+CD16high cells were higher or lower than the cut-off value. [file 12865_2020_375_MOESM2_ESM.pdf]

Fig S3

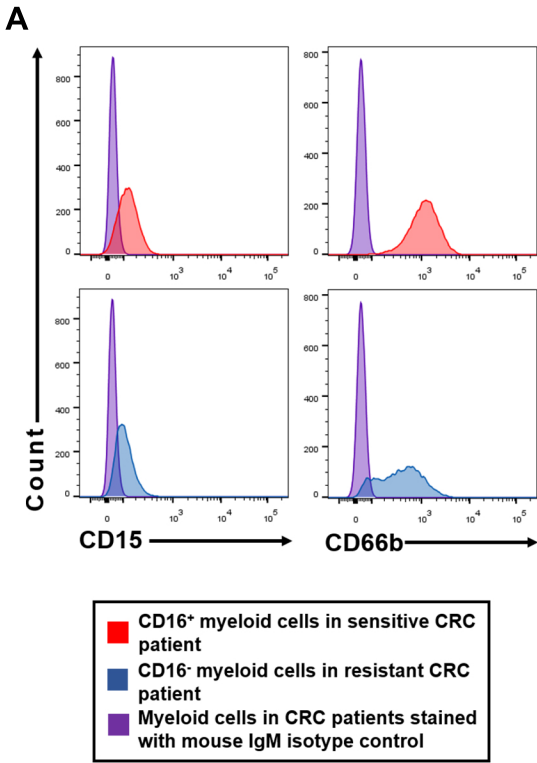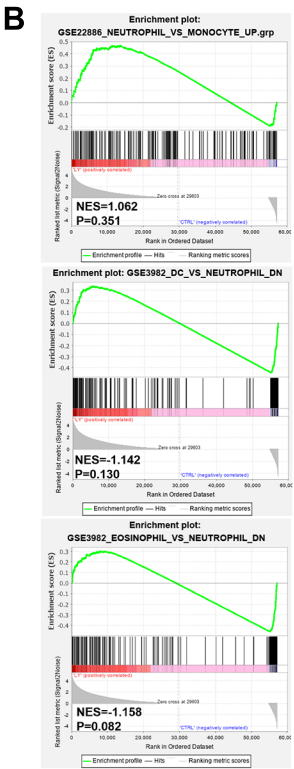

Supplement: Supplementary file 3 — Additional file 3: Figure S3. CD11b+CD16+ myeloid cells and CD11b+CD16− myeloid cells were neutrophils. (A) Peripheral venous blood from capecitabine-resistant and capecitabine-sensitive CRC patients after the therapy was collected. CD11b+CD16+ myeloid cells in sensitive patients and CD11b+CD16− myeloid cells in resistant patients were analyzed for their CD15 and CD66b expression. Meanwhile, a part of peripheral blood leukocytes from same patients were stained with mouse IgM isotype control. CD15 and CD66b expression were compared between cells stained with antibodies and that stained with isotype control. (B) The data of RNA sequencing was compared with published data of neutrophils using GSEA. Representative gene sets of neutrophil signature were shown. [file 12865_2020_375_MOESM3_ESM.pdf]
