# Supplementary material for: CD16 expression on neutrophils predicts treatment efficacy of capecitabine in colorectal cancer patients
Source: BMC Immunol. 2020 Aug 8;21:46. doi: 10.1186/s12865-020-00375-8 (PMC7414545; doi:10.1186/s12865-020-00375-8)
Supplement: Supplementary file 4 — Additional file 4: Table S1. Baseline characteristics of CRC patients in RNA sequencing. [file 12865_2020_375_MOESM4_ESM.pdf]

Table S1. Baseline characteristics of CRC patients in RNA sequencing.

Group	Number of Patients	Age	Sex	TNM Stage	Location	Diagnosis of Recurrence After Capecitabine Treatment
Resistant CRC patient	1	48	F	Ⅱ	Rectum	Yes
	2	67	M	Ⅲ	Colon	Yes
Sensitive CRC patient	3	55	M	Ⅱ	Rectum	No
	4	61	F	Ⅱ	Colon	No
