# Supplementary material for: CD16 expression on neutrophils predicts treatment efficacy of capecitabine in colorectal cancer patients
Source: BMC Immunol. 2020 Aug 8;21:46. doi: 10.1186/s12865-020-00375-8 (PMC7414545; doi:10.1186/s12865-020-00375-8)
Supplement: Supplementary file 5 — Additional file 5: Table S2. Gene set enrichment analysis of neutrophil signature. The data of RNA sequencing was compared with published data of neutrophils using GSEA. [file 12865_2020_375_MOESM5_ESM.pdf]

Table S2. Gene set enrichment analysis of neutrophil signature.

GeneSet	ES	NES	NOM p-val	FDR q-val	FWER p-val
GSE3982_DC_VS_NEUTROPHIL_DN	-0.44325554	-1.1419026	0.13043478	0.7566767	0.95
GSE3982_NEUTROPHIL_VS_BCELL_UP	-0.40136227	-1.0971215	0.1594203	0.8070399	0.98
GSE3982_NEUTROPHIL_VS_EFF_MEMORY_CD4_TCELL_UP	-0.50342894	-1.1381239	0.225	0.61691934	0.95
GSE22886_NAIVE_CD4_TCELL_VS_NEUTROPHIL_DN	-0.3980943	-1.0637071	0.3148148	0.80499357	0.99
GSE22886_NAIVE_CD8_TCELL_VS_NEUTROPHIL_DN	-0.40389994	-1.0748298	0.26229507	0.81070286	0.99
GSE22886_NAIVE_TCELL_VS_NEUTROPHIL_DN	-0.4247377	-1.1394007	0.17857143	0.65468645	0.95
GSE3982_DC_VS_NEUTROPHIL_LPS_STIM_DN	-0.44123843	-1.1699814	0.16666667	0.8963033	0.9
GSE3982_EOSINOPHIL_VS_NEUTROPHIL_DN	-0.45642194	-1.1579776	0.08163265	0.7221987	0.92
GSE3982_NEUTROPHIL_VS_CENT_MEMORY_CD4_TCELL_UP	-0.49643174	-1.1614217	0.16666667	0.77029866	0.91
GSE3982_NEUTROPHIL_VS_NKCELL_UP	-0.4798183	-1.1687933	0.1	0.7681386	0.9
GSE3982_NEUTROPHIL_VS_TH1_UP	-0.43271157	-1.1921971	0.06896552	0.66128355	0.87
GSE3982_NEUTROPHIL_VS_TH2_UP	-0.45457745	-1.196495	0.071428575	0.78912044	0.86
GSE22886_NAIVE_BCELL_VS_NEUTROPHIL_DN	-0.6152943	-1.22062	0.17073171	0.64763665	0.84
GSE22886_NEUTROPHIL_VS_MONOCYTE_UP	0.46898615	1.0624084	0.35135135	0.35135135	0.13
